# The story of artesunate–mefloquine (ASMQ), innovative partnerships in drug development: case study

**DOI:** 10.1186/1475-2875-12-68

**Published:** 2013-02-21

**Authors:** Susan Wells, Graciela Diap, Jean-René Kiechel

**Affiliations:** 1 Drugs for Neglected Diseases initiative (DNDi), Geneva, Switzerland

**Keywords:** Partnerships, Drug development, Artemisinin-based combination therapy, Artesunate, Mefloquine

## Abstract

**Background:**

The Drugs for Neglected Diseases *initiative* (DND*i*) is a not-for profit organization committed to providing affordable medicines and access to treatments in resource-poor settings. Traditionally drug development has happened “in house” within pharmaceutical companies, with research and development costs ultimately recuperated through drug sales. The development of drugs for the treatment of neglected tropical diseases requires a completely different model that goes beyond the scope of market-driven research and development.

Artesunate and mefloquine are well-established drugs for the treatment of uncomplicated malaria, with a strong safety record based on many years of field-based studies and use. The administration of such artemisinin-based combination therapy in a fixed-dose combination is expected to improve patient compliance and to reduce the risk of emerging drug resistance.

**Case description:**

DND*i* developed an innovative approach to drug development, reliant on strong collaborations with a wide range of partners from the commercial world, academia, government institutions and NGOs, each of which had a specific role to play in the development of a fixed dose combination of artesunate and mefloquine.

**Discussion and evaluation:**

DND*i* undertook the development of a fixed-dose combination of artesunate with mefloquine. Partnerships were formed across five continents, addressing formulation, control and production through to clinical trials and product registration, resulting in a safe and efficacious fixed dose combination treatment which is now available to treat patients in resource-poor settings. The south-south technology transfer of production from Farmanguinhos/Fiocruz in Brazil to Cipla Ltd in India was the first of its kind. Of additional benefit was the increased capacity within the knowledge base and infrastructure in developing countries.

**Conclusions:**

This collaborative approach to drug development involving international partnerships and independent funding mechanisms is a powerful new way to develop drugs for tropical diseases.

## Background

The Drugs for Neglected Diseases *initiative* was founded in 2003 as a collaborative, patients’ needs-driven, not-for-profit drug research and development (R&D) organization in order to develop new or improved treatments for neglected diseases, such as malaria, leishmaniasis, human African trypanosomiasis and Chagas disease. In 2011, DND*i* extended its activities to include projects on paediatric HIV and specific helminth infections. It operates with an alternative model [1] from that utilized by the pharmaceutical industry, where research and development is funded and mainly undertaken in-house with the aim of producing a profitable drug. As a product development partnership, DND*i*’s funding is provided by governments and charitable or philanthropic organizations to develop much needed anti-infectives of little commercial interest. The development model is dependent upon strong collaborations and partnerships worldwide, across academia, public health institutes, and the biotechnology and pharmaceutical industries.

The World Health Organization (WHO) estimates that 3.3 billion people were at risk of malaria in 2010, of which 1.2 billion living in the African and Southeast Asian regions were at the highest risk. Approximately 81%, or 174 million (113–239 million) cases were in the African region, with another 13% in the Southeast Asian region; the vast majority (91%) of deaths occur in the African region. Children under five years of age are the worst affected, accounting for approximately 86% of malaria deaths worldwide [2].

In the 1980s, there was a rise in resistance of malaria to chloroquine in many parts of Southeast Asia and South America, and emerging resistance along the east coast of Africa which led to the recommendation by the WHO in 2001 [3] to abandon the use of chloroquine worldwide and switch to using artemisinin-based combination therapy (ACT) as first-line treatment for uncomplicated *Plasmodium falciparum* malaria. Four different forms of ACT were recommended by the WHO: artemether/lumefantrine, artesunate + amodiaquine, artesunate + sulphadoxine/pyrimethamine and artesunate + mefloquine (for areas of low transmission).

Mefloquine (MQ) was originally discovered by the Walter Reed Army Institute of Research. However, although it was not covered by a patent, it was expensive to produce, and there was little initial interest in its development. The UNICEF-UNDP-World Bank-WHO Special Programme for Research and Training in Tropical Diseases (TDR) worked with the pharmaceutical company Hoffmann-la-Roche to develop a cheaper synthetic pathway, and sponsored more than 12 clinical trials in Latin America, Zambia and Thailand, leading to product registration in 1984 [4]. Initial successes in clinical trials carried out in Bangkok showed efficacy against multi-resistant *P. falciparum*[5], and in 1986 the combination of MQ with sulphadoxine/pyrimethamine (MSP) was 98% effective in treating 5,000 patients on the Thai-Burmese border [6]. However, *P. falciparum* resistance quickly developed, and in 1989 the MSP combination was abandoned [7].

Artemisia, or Qinghaosu, is a traditional Chinese herbal cure that has been used to treat malaria for over a thousand years. Isolated from the leaves of *Artemisia annua* (annual wormwood), it is a potent anti-malarial. Modern day semi-synthetic analogues have improved its solubility and absorption, and there are now a number of artemisinin derivatives available, including the water-soluble artesunate (AS), which forms part of the arsenal of combination treatments for malaria [8]. Artemisinin derivatives cause rapid clearance of parasites from the blood and a speedy resolution of symptoms, but as they are rapidly cleared from the system they are used in combination with a slower acting drug that kills the remaining parasites over three days of treatment.

Thailand was the first country to implement an artemisinin combination therapy (ACT) regime, following extensive use of MQ with AS along the Thai-Burma border in the early 1990s [9-11], leading to its adoption as national policy for the treatment of uncomplicated malaria in 1994, which continues to this day. Given the growing problem of delayed parasite clearance of *P. falciparum* malaria in Southeast Asia countries, indicative of artemisinin-resistant malaria, with early evidence confirmed at the Cambodia-Thailand border [12], WHO took the unprecedented step of requesting pharmaceutical companies to immediately halt the production of single-drug, artemisinin-containing tablets in order to protect this class of drugs [13]. Since 2006 WHO has recommended the use of artemisinin-based combination therapy to ensure high cure rates of *falciparum* malaria and to reduce the spread of drug resistance [14].

A search for suitable combinations of existing malaria drugs to control malaria resistance was carried out in 1998 by TDR, with support from the US Agency for International Development (USAID) and the Wellcome Trust. The resulting data analyses showed a combination of AS and MQ to be a good clinical option in Latin America and Southeast Asia. The WHO technical consultation on anti-malarial drug combination therapy in 2001 [3] suggested producing a fixed dosage formulation of ASMQ containing mefloquine at 8 mg/kg to be administered daily over three days [15], in order to optimize the use of co-formulations (AS+MQ). As there was a clear public health need but a lack of research groups willing to invest in its development, the Fixed-dose Artesunate-based Combination Therapy (FACT) consortium, born out of the initial partnership between DND*i* and TDR, undertook the development of ASMQ (and AS plus amodiaquine) fixed-dose combinations (FDC) [16,17]. FACT aimed to produce an affordable and widely available FDC that could be available to treat patients most affected by malaria whilst other potential drugs were still in early development.

## Case description

The FACT consortium was founded by DND*i* and TDR in 2002, and included the Brazilian government-owned pharmaceutical company, Farmanguinhos/Fiocruz, the Université Bordeaux Segalen, Universiti Sains Malaysia, Mahidol University and the Shoklo Malaria Institute in Thailand, the Centre National de Recherche et de Formation sur le Paludisme (CNRFP) in Burkina Faso and the University of Oxford as core members. Partnerships were set up with various industrial, academic, governmental and non-governmental organizations who carried out specific aspects of research, and an advisory committee made up of international experts met annually to advise and ensure all questions related to the worldwide treatment of malaria and the use of ACT were discussed. FACT members also carried out specific research in the development process.

Analytical and bio-analytical methods were developed by Universiti Sains Malaysia and subsequently transferred to Farmanguinhos/Fiocruz. Farmanguinhos performed the preformulation and formulation work required to scale up to industrial scale production. Quality control methods were developed and validated. The Université Bordeaux Segalen financed toxicology and genotoxicology studies performed by Unitox and Genotox in Brazil.

Initial trials with the FDC were conducted by clinical partners across Southeast Asia and Latin America.

A pioneer working relationship was developed with Brazilian regulatory authorities, the Agencia Nacional de Vigilancia Sanitaria (ANVISA), to prepare a dossier for submission for drug registration in the country. Consultation meetings were organized at different phases of the development on the way to the submission of the regulatory dossier (2005–2008).

In order to make the drug more easily accessible in Asia, a technology transfer agreement (2007–2009) enabled the drug manufacturing process to be transferred from its original manufacturer’s, the government-owned pharmaceutical company Farmanguinhos in Brazil, to Cipla Ltd, a generic pharmaceutical company in India. Cipla Ltd manufactured registration batches of the product in 2009, and a prequalification dossier was submitted to WHO in 2010, with members of the FACT team clarifying responses to enquiries.

## Discussion and evaluation

Artesunate and mefloquine are well known treatments for falciparum malaria in loose combination. Since 2001, the ASMQ combination has been one of the WHO-recommended forms of ACT for first-line treatment to provide adequate cure-rates and delay the development of resistance. DND*i* and partners decided to improve this therapy by developing a FDC of ASMQ, as the convenience afforded by reducing tablet numbers to once daily over three days is expected to lead to increased patient compliance and adherence. Such an undertaking required both pharmaceutical and industrial development as well as clinical studies. The subsequent technology transfer from Farmanguinhos, Brazil to Cipla Ltd, India facilitated access in endemic regions of Asia.

Development of a FDC of ASMQ has depended on close collaborations between industrial, academic, governmental and NGO research organizations, involving partnerships with and between developing countries, with independent funding provided by both public (EU’s INCODEV FP5, French AFD, Dutch DGIS, Spanish AECID, Swiss SDC, UK DFID and the European Commission’s EDCTP) and private (Médecins Sans Frontières, MSF) sources. The global cost of the development of ASMQ FDC, including pharmaceutical development costs, clinical studies, technology transfer and some implementation activities, is expected to amount to approximately 15 million Euros. This figure does not include in kind contributions by Farmanguinhos and Cipla Ltd, who contributed to the industrialization of the fixed dose combination.

### Pharmaceutical and preclinical development

The Universiti Sains Malaysia developed analytical and bio-analytical methods [18] for use in quality control and clinical pharmacological studies. The analytical techniques were transferred to Farmanguinhos/Fiocruz who formulated a fixed-dose combination product with appropriate stability and biopharmaceutical characteristics, together with a viable manufacturing process and the required validated control methods. Clinical batches were produced and toxicology, bio-equivalence, bio-availability and pharmacokinetic studies performed for inclusion in registration and prequalification files.

### Clinical development

Artesunate and MQ have been used in combination for over 20 years, involving more than 38,000 patients in 91 studies in 22 countries across Southeast Asia, the Western Pacific, Africa and Latin America [19]. The fixed dose combination of these has been demonstrated to be efficacious and safe for treating uncomplicated malaria in more than 25,000 patients in Thailand, Myanmar, India and in a large intervention study in Brazil (Table 1) [20-26]. Of these, the safety and efficacy study carried out on 500 patients in Thailand was pivotal, with efficacy of the FDC comparable to that of the loose tablet combination, better toleration and a lower incidence of vomiting. In addition, the Myanmar study (808 patients) compared the effectiveness of the four ACT FDCs recommended by WHO at the time and showed the ASMQ FDC to have the highest cure rate and the lowest rate of gametocyte carriage, providing the greatest post-treatment suppression of recurrent *falciparum* malaria and the most effective suppression of blood-stage *Plasmodium vivax* malaria [25].

**Table 1:** Key clinical studies

**Country**	**Study type**	**Phase**	**n**	**Primary objective**	**Major findings**
Thailand	HNV, PK and tolerability	1	24	PK and bio-availability of AS and MQ; administered separately and as a coformulation to healthy volunteers and patients	No difference was seen in the bio-equivalence of MQ in the two formulations; although a difference was seen for AS/DHA, this was not concluded to be clinically relevant [20]
	PK, efficacy and safety	2	50	Population PK model of new dosage regime of MQ with AS in loose combination over three days	Splitting the 25 mg/kg dose of MQ into three doses of 8 mg/kg improved oral bio-availability compared to the conventional split-dose regimen results. New regimen was well tolerated and with an equivalent therapeutic response [21]
	PK, efficacy and safety	2b	50	Safety and PK of ASMQ FDC vs non-fixed AS+MQ against multi-drug resistant falciparum malaria in adults	FDC well-tolerated with broadly similar PK profiles to non-fixed AS plus MQ [22]. The observed slowing of heart rate was best explained by malaria resolution [23]
	Efficacy and safety	3	500	ASMQ FDC with separate tablets in adults and children with uncomplicated multidrug-resistant falciparum malaria	Cure rates were 91.9% for the FDC after 63 days, with a lower incidence of vomiting compared to those in the loose tablets group [24]
Myanmar	Competitive Effectiveness	4	808	Effectiveness of five artemisinin combination regimens with or without PQ in uncomplicated falciparum malaria	ASMQ provided the greatest post-treatment suppression of malaria after 63 days, with the addition of PQ substantially reducing transmission potential of malaria [25]
India	Efficacy and safety	3	77	PCR corrected cure rate of ASMQ FDC in adult patients with uncomplicated falciparum malaria	Mean parasite clearance time of 48 hours
Brazil	Intervention study: PK Efficacy and safety	3b/4	23,845	Effectiveness of ASMQ FDC in reducing malaria transmission in the Juruá valley	Early detection of malaria and treatment with ASMQ FDC was feasible and efficacious, significantly reduced the incidence and morbidity of falciparum malaria [26]

Mefloquine has been associated with an increased incidence of nausea, vomiting, dizziness, dysphoria and sleep disturbance in clinical trials [27-47] and adverse reactions of varying severity have been experienced by a number of patients when taking AS and MQ in a loose combination or the FDC, primarily in the first 28 days of treatment. However, WHO [14] notes that mefloquine-related adverse effects are seldom debilitating and where this ACT has been deployed it has been well tolerated. There were no serious adverse effects reported in the Brazilian intervention study involving more than 23,000 patients, including children, confirming the safety of the combination. In addition, ASMQ FDC was designed to provide an optimized, short-term, gastro-intestinal tolerance, reducing the overall risk of vomiting. The risk of increased neuropsychiatric reactions following the use of MQ within 60 days of treatment is not an issue in Southeast Asia, Western Pacific and Latin America, areas with low to medium transmission.

**Figure 1:**
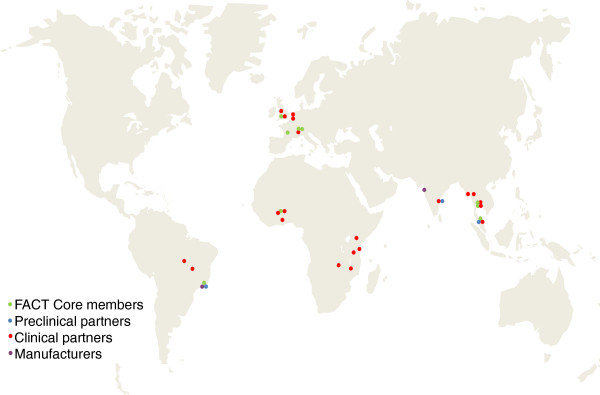
**International partners involved in the development of ASMQ FDC.** FACT Core Members: DND*i*, TDR of WHO, Farmanguinhos/Fiocruz (BR), Université Bordeaux Segalen (FR), Universiti Sains Malaysia (MY), Mahidol University (TH), Mahidol Oxford Tropical Medicine Research Unit, Shoklo Malaria Research Unit (TH), Centre National de Recherche et de la Formation sur le Paludisme (CNRFP) (BF), University of Oxford (UK). **Preclinical Development**: Universiti Sains Malaysia (MY), Farmanguinhos /Fiocruz (BR), Cipla Ltd (IN). **Clinical Development**: *Southeast Asia*: University of Oxford (UK), Mahidol University (TH), Mahidol Oxford Tropical Medicine Research Unit (TH), University Sains Malaysia (MY), Shoklo Malaria Research Unit (TH), Mae Sot Clinic (TH), Indian Council for Medical Research (IN), Médecins Sans Frontières, Medical Action Myanmar (MM); *Latin America*: Amazon Network for the Surveillance of Anti-malarial Drug Resistance (RAVEDRA)/Ministry of Health (BR), National Malaria Control Program Brazil; *Africa*: CNRFP (BF), Kenya Medical Research Institute (KEMRI) (KE), Institute of Tropical Medicine Anvers (BE), National Institute for Malaria Research (TZ), Kwame Nkrumah University (GH), Centre Muraz (BF), University of Malawi (MW), Tropical Medicines Research Centre (ZM), Royal Tropical Institute (KIT) (NL), Liverpool School of Tropical Medicine (UK), Ifakara Health Institute (TZ). **Manufacturers**: Farmanguinos/Fiocruz (BR), Cipla Ltd (IN).

The initial targeting of Southeast Asia and Latin America was based on the low to middle intensity transmission and multidrug resistance forms of malaria in these regions. Further studies involving ASMQ FDC are now ongoing with a number of partners in African countries (Figure 1; Table 2), including children and pregnant women, and patients with sickle cell anaemia, which will provide additional information for areas with a different epidemiological profile. A large-scale study in pregnant women is also ongoing in Thailand. Children and pregnant women are among the most vulnerable of those affected by malaria.

**Table 2:** Ongoing clinical studies

**Country**	**Study type**	**Phase**	**n**	**Primary objective**
Nigeria	Sickle cell proguanil *vs* AS+MQ *vs* SP+AQ	1/2	270	Safety and tolerability of bi-monthly intermittent preventive treatment with AS+MQ or sulphadoxine-pyrimethamine + amodiaquine compared to proguanil for prevention of malaria and related complications in patients with sickle cell anaemia
Amazon Basin	Efficacy	2/3	100	Evaluate the effectiveness of ASMQ FDC to treat uncomplicated falciparum malaria in the Juruá Valley
Burkina Faso	Pregnant women	2/3	48	PK of ASMQ FDC in pregnant women
Burkina Faso, Ghana, Malawi and Zambia	Pregnant women: ASMQ *vs* DHA-PQ *vs* ASAQ *vs* AL	3	3500	Efficacy and safety of four ACTs (artemether-lumefantrine, amodiaquine-artesunate, mefloquine-artesunate and dihydroartemisinin-piperaquine) in pregnant women with *P. falciparum* malaria
Tanzania, Burkina Faso and Kenya	Efficacy, safety and PK in children ASMQ *vs* AL	4	940	Efficacy, safety and population pharmacokinetics ASMQ FDC in African children *vs* artemether-lumefantrine
Thailand	Pregnant women: ASMQ *vs* DHA-PQ *vs* AL	3	1,000	Randomized trial of three ACT for malaria in pregnancy (DMA)
Brazil	Efficacy and safety *P. vivax*: ASMQ+PQ *vs* CQ+PQ *vs* AL+PQ	3	264	Efficacy and safety for treating *P. vivax*: ASMQ+PQ *vs* CQ+PQ *vs* AL+PQ

ASMQ FDC could also play a role within the strategy of multiple first-line therapies to delay emergence and spread of disease where, in areas of high transmission, repeat treatments are likely to be necessary within 60 days of the initial treatment. A recent model predicts that in areas with high transmission, combining a gametocytocidal artemisinin derivative with a longer-acting partner drug will protect patients from re-infection and reduce transmission more effectively than some common currently used ACT regimens which are gametocyte-killing but short-acting [48].

*Plasmodium vivax* is the second most important species causing human malaria, accounting for about 40% of malaria cases worldwide [14]. It is the dominant malaria species outside Africa, prevalent in endemic areas in Asia, Central and South America, Middle East and Oceania but rare in Africa. WHO recommends treating uncomplicated *vivax* malaria with chloroquine in combination with primaquine, where chloroquine is effective. However, in areas of chloroquine resistance an appropriate ACT (but not artesunate-sulphadoxine/pyrimethamine) combined with primaquine is to be used, particularly where ACT is first line therapy for *P. falciparum*, although primaquine is counter-indicated with severe glucose-6-phosphate dehydrogenase (G6PD) deficiency. Experience indicates that it is likely that primaquine combined with ASMQ will result in high cure rates against vivax blood stage infection and relapse [49]. In Brazil, the Tropical Pathology Research Institute (IPEPATRO) is studying the efficacy of ASMQ FDC in treating *P. vivax* malaria compared to chloroquine or artemether-lumefantrine when each is administered with primaquine.

### Technology transfer

Within the FACT consortium, Farmanguinhos/Fiocruz was the first manufacturer of ASMQ FDC. The technology transfer of ASMQ FDC between Farmanguihos/Fiocruz in Brazil and Cipla Ltd (2007–2009) in India was undertaken to facilitate access to ASMQ in Southeast Asia. There were some problems encountered during this first-time process. A halt in the production of MQ API by the original manufacturer led to significant delays whilst a new manufacturer was identified and the product evaluated. There were differences in the particle size and flow compatibility of this replacement API compared with the original product necessitating changes to the manufacturing process, which required some time. In addition, Cipla’s equipment differed from that used by Farmanguinhos, requiring further modifications to the process. However these improvements ultimately resulted in faster tablet manufacture of ASMQ to GMP-standards. With the resolution of these problems the transfer was completed in 2010, and as such it is a prime example of a successful south-south technology transfer.

With Cipla Ltd in charge of manufacturing in Asia, together with Indian registration granted in November 2011, the supply of ASMQ in Asia and in other parts of the world is ensured at pre-agreed prices [50].

### Product registration and prequalification

DND*i* worked with the Brazilian regulatory authorities, the Agencia Nacional de Vigilancia Sanitaria (ANVISA), in order to register ASMQ FDC for use in the country. In late 2006, a new piece of Brazilian registration incorporated an expedited review process for the drugs to treat neglected diseases. ASMQ FDC was granted Brazilian registration approval in March 2008 and adopted as treatment policy by the Ministry of Health as an alternative to first-line treatment for uncomplicated *P. falciparum* malaria [51].

On 29 March, 2012, the Malaysian National Pharmaceutical Control Bureau of the Ministry of Health approved the use of ASMQ FDC for the treatment of acute uncomplicated malaria resulting either from *P. falciparum* mono-infection or mixed infections with *P. vivax*. This approval is a step for ASMQ FDC within the ASEAN (Association of Southeast Asian Nations) Harmonization Scheme between health authorities, and as Malaysia is a Pharmaceutical Inspection Convention country, it should facilitate registration in the other countries. DND*i* is coordinating the review of ASMQ with ASEAN regulatory authorities to obtain drug registration, which will in turn lead to adoption by malaria control programmes. The registration of ASMQ FDC is underway in countries in Southeast Asia and the Western Pacific where AS+MQ is part of a national policy for uncomplicated malaria [52].

WHO pre-qualified the Cipla ASMQ FDC product in September 2012. This recognition of a high-quality product will enable procurement agencies to bulk-buy goods for use in resource-poor settings. A recent analysis found that a large percentage of anti-malarial drugs in Asia and sub-Saharan Africa are either fake or of inferior quality, leading to vulnerable populations receiving poor treatment and adding to the threat of resistance developing [53].

### Working with a virtual model

The DND*i* model works through collaboration and cooperation between international partners from a wide range of research settings. As such it has required the management of collaborators spread over different research centres across Europe, USA, Asia, South America and Africa. This has led to management problems not necessarily encountered in a conventional industrial setting, notably in terms of communication between the various project groups. Bi-annual project meetings were held in order to communicate results effectively between all project partners, interspersed with local research group meetings held at the relevant sites. This dispersal of knowledge and personnel meant that it took one to two years before a team could be sufficiently well established to work together efficiently and effectively, and adaptation to differing political and cultural influences was required. However, the overall belief in the value of the project, together with a desire to succeed, was highly motivating for all involved, and much was achieved.

This virtual model gave rise to some complications which would have been simpler to resolve in a traditional pharmaceutical setting, based on existing infrastructure and know-how. For example, in addition to those problems described above relating to the production of mefloquine API, another change of manufacturer had to be managed very late in development due to a halt in production of artesunate API by the selected supplier. It was essential to demonstrate the identity of the new API selected and its GMP status, as well as ensuring the Drug Master File (DMF) acceptability by regulatory authorities and WHO. In both cases, as the FACT partnership did not represent a potential big customer this made negotiations more difficult and required good will from the suppliers, who became, to some extent, members of the partnership. These API changes had an impact on the development and registration timelines. A further complication was more typical of those experienced by international organizations. The partner in Brazil, Farmanguinhos, developed the FDC according to local specifications, or to the specification in force in production. This required vigilance and adaptation, with additional analysis and documentation subsequently necessary in order for the manufactured product to fulfill development and quality specifications for worldwide use.

Finally, working with a number of different partners magnified the effects of natural turnover in personnel, at different levels. There was occasionally a risk of lack of continuity in vision which required a prolongation of project management beyond the initial years of early development, and the sustained support from the executive level management of the participating partners.

### Capacity building

Partnerships have been set up with and between developing countries, where there has been the added benefit of building capacity of resources and knowledge in countries in South America, Southeast Asia and Africa. This in turn may lead to increased product uptake, and to local scientists and pharmaceutical firms pursuing follow-on innovations that could address local access issues [54].

Farmanguinhos/Fiocruz has acquired much useful knowledge and experience in its involvement with the project. ASMQ FDC was the first new product fully developed by the company, taking it through from development of the manufacturing and control processes, validation of control methods and manufacturing processes, industrial scale up and production, compilation of the regulatory file and on to successful product registration in Brazil. One employee received training in the preparation of the analytical methods related to AS at Universiti Sains Malaysia. This knowledge was passed on to Farmanguinhos/Fiocruz, as an enabling step in the control of the production process and product development. Other members of the Brazilian development team received training in toxicology studies.

During the course of this project, team members at the University Sains Malaysia learned how to perform bio-analytical work under GLP conditions, and those involved in clinical development acquired a better understanding of the requirements in developing a drug to ICH standards for regulatory approval. In India, staff involved in clinical trials were trained to GCP standards, including training in maintaining correct documentation and the completion of case record forms.

## Conclusions

Artesunate and MQ in combination have been adopted as first-line treatment of uncomplicated malaria in several countries in Southeast Asia (Cambodia, Malaysia, Thailand and Myanmar) and South America (Bolivia, Peru, Brazil and Venezuela) [52]. In Brazil, where ASMQ FDC is produced by the government-owned pharmaceutical company Farmanguinhos/Fiocruz, the government provides free treatment to patients. Cipla Ltd has committed to making their product available at pre-agreed, affordable prices.

DND*i* and its partners are working towards lowering the price of ASMQ in the future. The Medicines for Malaria Venture (MMV) and DND*i* have had a long-standing relationship, which ranges from reciprocal access to high-throughput drug screening results through to joint support of clinical studies. MMV has led a collaborative effort to develop a low-cost process for MQ, together with Development Chemicals Ltd and Creative Chemistry, UK-based process chemistry specialists. As part of its work on developing a single isomer of MQ [55], MMV has developed a significantly cheaper manufacturing process, resulting in a reduction in bulk drug from 1,100 USD/kg to 400 USD/kg.

The development of a semisynthetic artemisinin, prepared from a dihydro-artemisinic acid (DHAA) precursor synthesized by *Saccharomyces cerevisiae*[56,57], will be at the lower end of the AS pricing band, so ensuring availability at an optimized cost.

The availability of low-cost versions of AS and MQ will ultimately increase access to the FDC treatment. Pragmatic strategies such as the Affordable Medicines Facility for malaria (AMFm) to subsidize anti-malarials and demand forecasting (PAHO Strategic Fund) should also facilitate access to ACT at affordable prices.

With several quality-assured types of ACT available in the market and very promising combinations in the pipeline, more efforts should be made to use all the available tools towards better control and elimination of malaria.

DND*i* was set up as a not-for-profit organization in 2003 in order to address the pressing need for effective treatments for patients with the most neglected tropical diseases, as one of the first Public Private Partnerships (now known as Product Development Partnerships). The development of a FDC of ASMQ was one of its initial projects, and the lessons learnt have had an impact on its approach to drug development which will be applied to the management of other diseases across its portfolio and will require new and different partners according to the development stage of the candidate drugs. Following the successful development of two treatments for malaria (ASMQ and artesunate-amodiaquine), DND*i* will finalize the transfer of its malaria treatments to partners by 2014, in line with its recently revised business plan.

This collaborative approach to drug development enabled by independent funding mechanisms and involving partners from across the globe and from a variety of research settings, is a powerful new way to develop drugs for tropical diseases, so making new treatments available to patients in resource-poor settings.

## Abbreviations

ACT: Artemisinin-based combination therapy; ANVISA: Agencia Nacional de Vigilancia Sanitaria; AS: Artesunate; ASEAN: Association of Southeast Asian Nations; CNRFP: Centre National de Recherche et de Formation sur le Paludisme; DNDi: Drugs for Neglected Diseases *initiative*; FACT: Fixed-dose Artesunate-based Combination Therapies; FDC: Fixed dose combination; IPEPATRO: Research Institute of Tropical Pathology; MMV: Medicines for Malaria Venture; MQ: Mefloquine; MSF: Médecins Sans Frontières; PAHO: Pan American Health Organization; TDR: UNICEF-UNDP-World Bank-WHO Special Programme for Research and Training in Tropical Diseases; WHO: World Health Organization.

## Competing interests

The authors declare that they have no competing interests.

## Authors’ contributions

SW wrote the initial manuscript. GD and JRK made substantial contributions and helped to finalize the manuscript. All authors read and approved the final manuscript.
